# Unraveling the epigenetic fabric of type 2 diabetes mellitus: pathogenic mechanisms and therapeutic implications

**DOI:** 10.3389/fendo.2024.1295967

**Published:** 2024-01-22

**Authors:** Cham Jazieh, Tarek Ziad Arabi, Zohaib Asim, Belal Nedal Sabbah, Aljohara Waleed Alsaud, Khaled Alkattan, Ahmed Yaqinuddin

**Affiliations:** College of Medicine, Alfaisal University, Riyadh, Saudi Arabia

**Keywords:** epigenetics, diabetes mellitus, type 2 diabetes complications, obesity, insulin resistance, beta-cell dysfunction, cellular senescence, mitochondrial dysfunction

## Abstract

Type 2 diabetes mellitus (T2DM) is a rapidly escalating global health concern, with its prevalence projected to increase significantly in the near future. This review delves into the intricate role of epigenetic modifications - including DNA methylation, histone acetylation, and micro-ribonucleic acid (miRNA) expression - in the pathogenesis and progression of T2DM. We critically examine how these epigenetic changes contribute to the onset and exacerbation of T2DM by influencing key pathogenic processes such as obesity, insulin resistance, β-cell dysfunction, cellular senescence, and mitochondrial dysfunction. Furthermore, we explore the involvement of epigenetic dysregulation in T2DM-associated complications, including diabetic retinopathy, atherosclerosis, neuropathy, and cardiomyopathy. This review highlights recent studies that underscore the diagnostic and therapeutic potential of targeting epigenetic modifications in T2DM. We also provide an overview of the impact of lifestyle factors such as exercise and diet on the epigenetic landscape of T2DM, underscoring their relevance in disease management. Our synthesis of the current literature aims to illuminate the complex epigenetic underpinnings of T2DM, offering insights into novel preventative and therapeutic strategies that could revolutionize its management.

## 1 Introduction

Type 2 diabetes mellitus (T2DM) is a persistent metabolic condition associated with increasing concentrations of glucose in the blood characterized by impaired insulin action and/or inadequate insulin secretion. The global prevalence of T2DM has been rising quickly. A study by Saeedi et al. has projected the global incidence of T2DM to reach 8% by 2030 and 8.6% by 2045 (1). Therefore, there is a critical need for preventative measures to reduce the global health burden of the disease.

Epigenetics is a field that describes functional changes to the genome without changes to the nucleotide sequences of the deoxyribonucleic acid (DNA) (2). Several epigenetic modifications have been identified. The most commonly studied mechanism is DNA methylation, a crucial epigenetic modification, primarily functions to regulate gene expression by adding methyl groups to cytosine residues in DNA, often leading to the suppression of gene activity and affecting cellular differentiation, development, and disease pathogenesis (3). This process almost always occurs in CpG regions, where 70% of methylation events occur (4). Epigenetic modifications encompass a diverse range of mechanisms that regulate gene expression at the chromatin level. One significant type of epigenetic modification is histone acetylation. This process involves the addition of an acetyl group to the lysine residues of histone proteins, an action catalyzed by enzymes known as lysine acetyltransferases (5). Histone acetylation plays a crucial role in modulating the accessibility of chromatin and thereby influencing gene transcription. Beyond acetylation, the chromatin landscape is shaped by various other epigenetic modifications, including methylation, phosphorylation, ubiquitination, and sumoylation, each contributing uniquely to the regulation of gene expression.

Additionally, non-coding ribonucleic acids (ncRNAs) represent another essential facet of epigenetics (3). These RNA molecules, which are transcribed but not translated into proteins, are involved in genetic silencing. Beyond this, emerging evidence highlights the role of ncRNAs in orchestrating epigenetic modifications, further underscoring their significance in gene regulation (3, 6).

In addition to these mechanisms, the epitranscriptome represents a burgeoning area of interest in epigenetics, particularly in its relation to T2DM (7). The epitranscriptome involves modifications to RNA molecules, such as methylation, that do not alter their primary sequence but can significantly impact RNA stability, localization, and translation (8). These RNA modifications are akin to epigenetic changes as they regulate gene expression post-transcriptionally and are dynamically reversible (8). Such epitranscriptomic modifications offer another layer of genetic regulation and have been increasingly implicated in the pathogenesis of metabolic disorders like T2DM (8, 9).

Data have suggested that these epigenetic modifications are essential to the development of several diseases (10). Among these diseases, recent epigenetic studies have highlighted the role of epigenetic modifications in the pathogenesis of T2DM (11–13). Additionally, these mechanisms could be used to predict the risk of T2DM and, potentially, as therapeutic targets (14–16). The association between epigenetic modifications and T2DM is subject to ongoing debate. This uncertainty largely stems from the limited scope of existing literature and research in this area. As a result, establishing a definitive causal relationship between epigenetic changes and the development of T2DM remains a complex and unresolved issue (17, 18). However, a recent study by Juvinao-Quintero et al. identified a causal effect of the 24-dehydrocholesterol reductase gene *DHCR24* (a gene linked to lipid metabolism) methylation in T2DM risk (11). Therefore, studying and targeting the epigenetics associated with T2DM could open the door for preventative and therapeutic measures.

In this article, we synthesize the latest literature describing the role of epigenetics in various aspects of T2DM pathogenicity, including obesity, insulin resistance (IR), β-cell dysfunction, cellular senescence, and mitochondrial dysfunction. Then, we describe the epigenetics mechanisms through which exercise and dietary choices influence the risk of T2DM. Finally, we also describe the role of epigenetics in T2DM-related complications, including atherosclerosis, diabetic retinopathy, neuropathy, and cardiomyopathy.

## 2 Epigenetics in obesity

Obesity has been steadily increasing in the past decades, reaching a global prevalence rate of 14% in 2019 (19). Obesity is associated with several pathologies, including ischemic heart disease, non-alcoholic fatty liver disease, chronic kidney disease, several cancers, and T2DM (19–22). The mechanisms linking obesity and T2DM are well-established and have been reviewed extensively (23–25).

Epigenetic modifications have been shown to play a significant role in the development of obesity. Xu et al. conducted a genome-wide methylation on blood samples from 48 obese and 48 lean individuals aged 14-20 years (26). The authors identified more than 20,000 differentially methylated (DMC) and differentially variable (DVC) CpG sites associated with obesity status. Both DMC and DVC sites were able to predict obesity case-control status in the cohort (26). Lastly, genes containing DMCs and DVCs showed significant enrichment of genes associated with obesity-related disorders, including hypertension, dyslipidemia, and T2DM (26). Dagaard et al. uncovered a pivotal epigenetic mechanism that triggers obesity (27). They identified a Trim28-dependent network, which operates in a non-Mendelian, “on/off” manner, fundamentally influencing body weight distribution. This network is characterized by the decreased expression of key imprinted genes, including Nnat, Peg3, Cdkn1c, and Plagl1. Intriguingly, manipulating these genes can replicate the observed bi-stable, stochastic obesity phenotype (27). Their findings not only shed light on the enigmatic heritability of obesity but also highlight the potential for discrete polyphenism in obesity’s genetic underpinnings, both in mice and humans. Dick et al. identified five CpG cites associated with increased body mass index (BMI); among which, three are located within *HIF3A* (28). *HIF3A* is a transcription factor for hypoxia inducible factor-3 alpha responsible for regulating the body’s response to hypoxia (2). More recently, Janjanam et al. found that DMC sites on *NANOS1* and *SOX14* in newborns can modulate the risk of increased body weight at later stages of life (29). Methylation of specific CpG sites is not only associated with increased risk of obesity but can result in decreased risk of obesity as well. For example, Huang et al. found that a 1% increase in methylation in *TAOK3* decreased the odds of child obesity by 0.91 (30). The *TAOK3* gene encodes the STE20-type protein kinase TOAK3, which exacerbates lipid storage and oxidative/endoplasmic stress in hepatocytes (31).

**Figure 1:**
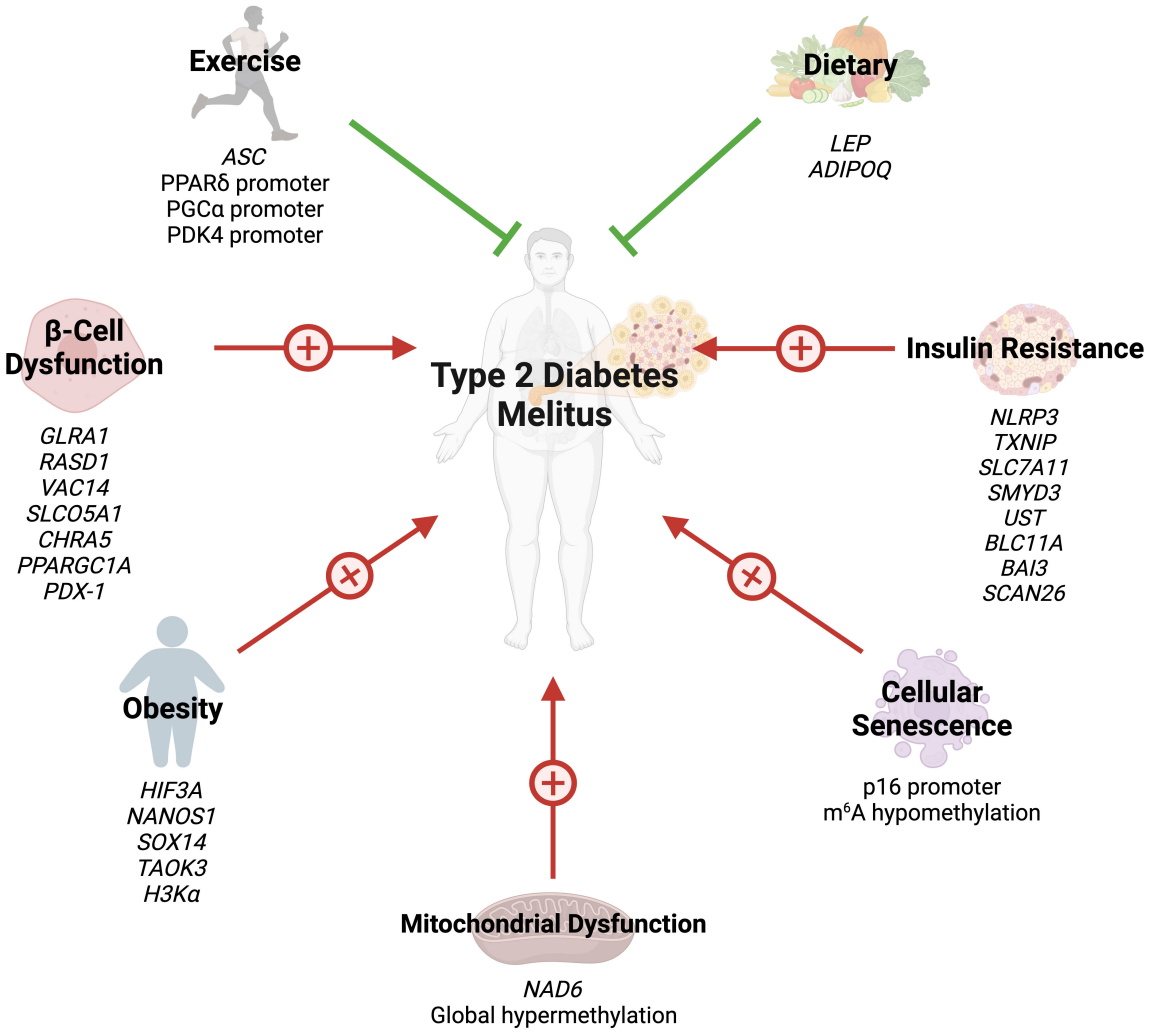
Several epigenetic modifications can modulate the risk of T2DM through obesity, insulin resistance, beta-cell dysfunction, cellular senescence, mitochondrial dysfunction, exercise, and dietary choices.

Several studies have also highlighted the role of histone acetylation in obesity. Hypertrophied adipocytes, the main cause of obesity, exhibit increased acetylation of histone H3 at lysine 9 and global hypomethylation of DNA (32). A study by Pessoa Rodrigues et al. studied the role of the histone H4 lysine 16 acetyltransferase MOF in metabolic regulation (33). Although *Mof* depletion resulted in resistance to weight gain, these modifications led to an insulin-glucose imbalance, resulting in T2D predisposition. MOF was also found to promote the proliferator-activated receptor-γ (PPARγ) and downstream transcriptional signals. Additionally, treatment with the PPARγ agonist thiazolidinedione restored glucose uptake in *Mof-*deficient mice (33). Collectively, these studies indicate that epigenetics play a key role in the development of obesity and obesity-related diseases (**Figure 1**).

## 3 Epigenetics in insulin resistance and β-cell dysfunction

The cornerstones of T2DM pathogenesis are IR and β-cell dysfunction, resulting in reduced insulin secretion (34). Understanding the various pathways that contribute to beta cell homeostasis and failure is essential for determining the pathophysiology of T2DM. β-cells of the pancreas synthesize insulin and store insulin. Through the action of the glucose transporter GLUT2, glucose enters β-cells where it is generates adenosine triphosphate (ATP) through the process of glycolysis. When the ATP/adenosine diphosphate ratio rises in hyperglycemic states, ATP-operated potassium channels are closed, depolarizing the cell membrane and opening voltage-gated calcium channels. Calcium influx triggers insulin release from secretory granules, which in turn regulates glucose levels in physiologic conditions (35).

IR is often the first trigger of T2DM and is initially offset by marked insulin release (36). However, increased insulin demands eventually lead to β-cell failure and T2DM progression. The role of epigenetics in both IR and β-cell failure has been described in the literature. Global DNA methylation in lymphocytes and visceral adipose tissue positively correlates with IR (37). On the other hand, a cross-ancestry epigenome-wide association study identified five CpG sites inversely associated with IR: three in *TXNIP*, one in *SLC7A11*, and another in *ZSCAN26* (38). *TXNIP* encodes for thioredoxin-interacting protein (TXNIP) which promotes β-cell apoptosis in hyperglycemic states (39). TXNIP depletion has been found to promote Akt/Bcl-xL pathways and β-cell mass, hence protecting against T2DM in mice (40). Zhang et al. compared DNA methylation in insulin sensitive and IR obese women (41). The authors identified four significant DMC sites: *SMYD3, UST, BCL11A*, and *BAI3*, which are involved in glycosaminoglycan and chondroitin/dermatan sulfate synthetic processes.

β-cell mass is reduced by up to 60% in T2DM (42), and loss of cellular function further progresses the condition (43). As previously described, several pathways regulating β-cell mass can be modified by epigenetics (38). The insulin promoter gene is methylated in human pancreatic islet cells of T2DM patients (44). Glycated hemoglobin levels correlate both positively and inversely with methylation of several CpG sites in the same patients (44). Additionally, *GLRA1, RASD1, VAC14, SLCO5A1*, and *CHRNA5* genes in human islet cells exhibit DNA methylation changes after exposure to high glucose levels (45). Similarly, DNA methylation of PPARγ *PPARGC1A* gene promoter in islet cells is near double in T2DM patients compared to their non-diabetic counterparts (46).

The transcription factor pancreatic duodenal homeobox-1 (PDX-1) plays a key role in pancreatic development and regulates mature islet cell physiology (47). Knockout of the PDX-1 gene results in failure of pancreatic bud formation and mice death from hyperglycemia (47). Epigenetic modifications of *PDX-1* in pancreatic cells have been extensively studied in T2DM. *PDX-1* methylation is significantly increases after high glucose exposure in pancreatic islet cells (45). Additionally, *PDX-1* methylation is significantly greater in T2DM pancreas cells (48).

β-cell malfunction and apoptosis are strongly linked to inflammation, especially low-grade chronic inflammation (49). Oxidative stress, which is characterized by an imbalance between the production of reactive oxygen species (ROS) and antioxidant defense systems, has a significant impact on β-cell failure. In addition to causing β-cell death and disrupting signaling pathways, ROS can reduces insulin secretion and predisposes patients to T2DM (50). The epigenetic link between inflammation and β-cell is understudied; however, emerging evidence has highlighted the role of epigenetics in this relationship. For example, IL-1β induces abnormal DNA methylation of islet cells, and treatment with methyltransferase inhibitors partially reverses IL-1β-induced β-cell dysfunction (51, 52).

MicroRNAs (miRNAs) are pivotal in the epigenetic interplay between inflammation and β-cell function, a critical facet in the pathogenesis of T2DM. The deregulation of specific miRNAs, such as miR-126 and miR-146a, which are notably downregulated in T2DM patients, exemplifies this connection (12). The alteration in the expression levels of these miRNAs can be attributed to epigenetic modifications, such as DNA methylation and histone modifications, which affect the transcription and stability of miRNA genes. For instance, miR-126, which has demonstrated high diagnostic accuracy for T2DM with a sensitivity of 91% and specificity of 97%, is subject to such epigenetic regulation (12).

Moreover, miR-375, predominantly expressed in the human pancreas, is regulated epigenetically through promoter methylation and interactions with circular RNAs (53). This miRNA, a key regulator of inflammation, has been implicated in various diseases including Grave’s disease, type 1 diabetes mellitus, and inflammatory bowel disease, and its levels are associated with insulin resistance markers in obese children (53, 54).

Additionally, miR-200 plays a proinflammatory role in vascular smooth cells of diabetic mice and is involved in β-cell damage and enhanced insulin resistance, with its effects being mediated through epigenetic modulation of signaling pathways (55–57). Furthermore, the knockdown of miRNAs such as -24, -26, -182, and -148 leads to a decrease in insulin promoter activity and insulin mRNA expression, further illustrating the epigenetic influence on miRNA-mediated regulation of β-cell function (58).

Hence, miRNAs serve not only as key components in the inflammatory response associated with T2DM but also as integral players in the epigenetic regulation of genes crucial for insulin secretion and β-cell function. Their potential utility as diagnostic and therapeutic markers in T2DM underscores the importance of understanding their epigenetic regulation mechanisms. The role of epigenetics in IR and β-cell failure is summarized in (**Figure 1**).

## 4 Epigenetics and cellular senescence in T2DM

Cellular senescence is a cellular hallmark of aging and is defined by irreversible cell cycle arrest due to telomere attrition (21). Cell senescence results in rapid development of age-related comorbidities and is associated with several conditions, including obesity, chronic kidney disease, and cancer (21, 59). Cellular senescence is mediated by upregulation of several cell cycle inhibitors, including p16^INK4a^, p15^INK4b^, p19^ARF^, and p21^CIP^ (60). Markers of cellular senescence are significantly increased in T2DM β-cells (61). Additionally, glycated hemoglobin, a marker of long-term diabetic control, is negatively correlated with telomere length in T2DM patients (62). Global DNA methylation of β-cells increases with age (63). Specifically, promoters of β-cell proliferation become methylated with increasing age (63).

Studies assessing the role of epigenetic modifications of cellular senescence markers in T2DM are rare. N^6^-methyladenosine (m^6^A) refers to methylation of adenosine at the nitrogen-6 position and is the most common internal messenger RNA modification in humans (64). Alterations in m^6^A promote ROS, telomere shortening, DNA damage, and pro-inflammatory functions, eventually leading to cellular senescence (65). T2DM results in m^6^A hypomethylation of genes involved in cell cycle progression, insulin release, and several insulin signaling pathways (66). Reduced m^6^A in β-cells results in cell cycle arrest and impairs insulin release via decreased AKT phosphorylation and PDX1 protein levels (66). This modification also enhances insulin messenger RNA translation, and depletion has been associated with T2DM-like states (67). Dietary changes can also hinder cellular senescence through epigenetic changes. Li et al. demonstrated that glucose restriction decreases p16 expression by exerting changes in histone acetylation and p16 promoter methylation (68). Collectively, these findings reveal that epigenetics regulate cellular aging in T2DM (**Figure 1**). However, further studies are needed to identify additional epigenetic changes in T2DM-associated cellular senescence.

## 5 Epigenetics and mitochondrial dysfunction in T2DM

Due to their key role in metabolic functions, it is unsurprising that mitochondrial dysfunction is a common characteristic among T2DM. T2DM and obesity patients have less mitochondria compared to their lean, non-diabetic counterparts (69). T2DM patients also exhibit significant reductions in electron transport chain activity (69). Furthermore, T2DM mitochondria demonstrate no response to insulin stimulus in several studies (70, 71). Mitochondria are the primary sources of ROS, which retards GLUT4 translocation and insulin signaling pathways (72, 73).

Recently, studies have shed light on the role of epigenetic modifications of mitochondrial DNA (mtDNA) in the context of T2DM. mtDNA is hypermethylated by hyperglycemic conditions (74). NADH-dehydrogenase-6 (NAD6), the only protein encoded by mtDNA L-strand, is greatly reduced in obese and T2DM populations, as a result of hypermethylation of *NAD6* (75). Hypermethylation is promoted by free fatty acids which induce DNA methyltransferase-1 mitochondrial translocation via AMPK activation (75). Inhibiting NAD6 results in systemic IR, and DNA methyltransferase-1 inhibition exerts opposite effects and improves insulin sensitivity in mice (75). mtDNA methylation results in capillary cell apoptosis and treatment with a DNA methyltransferase-1 inhibitor blunts these effects in diabetic retinopathy (74). These findings demonstrate that mtDNA epigenetic modifications can play a crucial role in the development of IR (**Figure 1**).

## 6 Epigenetic modifications related to exercise and diet

Emerging evidence has highlighted the effects of exercise on epigenetic modifications, specifically DNA methylation (**Figure 1**). Although several cross-sectional studies have identified epigenetic modifications related to exercise, their findings are greatly varying and contradictory (76). However, interventional studies have provided more clarity. Barrès et al. studied the effects of acute exercise on DNA methylation in human skeletal muscles (76). Acute exercise induced hypomethylation of PGCα, PDK4, and PPARδ promoters, resulting in increased expression of the proteins in a dose-dependent manner. Similar results can be seen in chronic exercise, which promotes *PDK4* expression in normal patients, but not diabetics (77). Chronic exercise also enhances *ASC* gene methylation, which is responsible for IL-1β and -18 production (78).

Dietary choices can also exert their impact on the epigenetics of T2DM. Nilsson et al. revealed DNA hypomethylation with concurrently reduced folate levels in T2DM levels (79). The authors hypothesized that reduced folate levels could lead to these epigenetic changes. Fasting has been shown to alter the epigenetic profile of humans. Hjort et al. found that 36 hour fasting increases methylation of *LEP* (leptin) and *ADIPOQ* (adiponectin) in the subcutaneous adipose tissue of patients with normal birth weight (80). The choice of dietary fat type also plays a great role in epigenetic modifications. Alexander et al. conducted a randomized, controlled trial where patients were either given excessive saturated fatty acids or polyunsaturated fatty acids (81). The methylation of 4875 CpG sites was affected differently between the two arms, and the mean methylation of 1444 genes in adipose tissue was altered by overeating. Finally, the baseline methylation of 12 CpG sites (9 genes) was associated with the degree of weight gain during the trial (81). Collectively, these findings demonstrate that lifestyle factors modify the epigenetic profiles of humans, possibly preventing or promoting the development of T2DM.

## 7 Epigenetic modifications in the complications of T2DM

**Figure 2:**
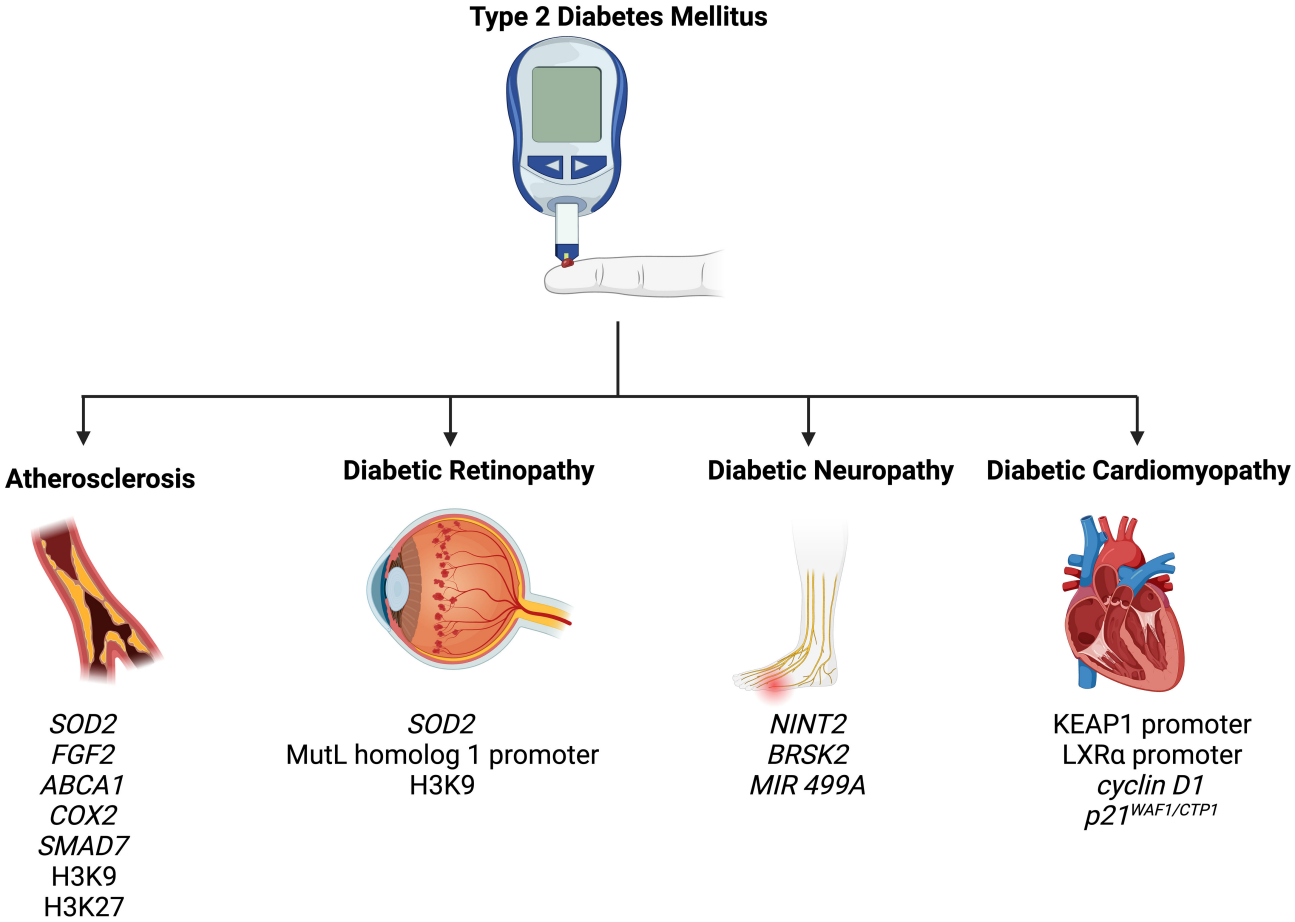
Epigenetic modifications play a role in the pathogenesis of T2DM-related complications, including atherosclerosis, diabetic retinopathy, diabetic neuropathy, and diabetic cardiomyopathy.

T2DM is associated with a wide spectrum of complications, including retinopathy, nephropathy, neuropathy, atherosclerosis, and cardiomyopathy (82). In the following section, we summarize how epigenetic mechanisms promote and modify T2DM-related complications (**Figure 2**).

### 7.1 Atherosclerosis

Atherosclerosis is an extremely common complication of T2DM, presenting in nearly 30% of patients (83). Dyslipidemia, hyperglycemia, and chronic inflammation collectively play major roles in atherosclerotic plaque formation in T2DM (84). Aberrant methylation of several genes, including *SOD2, FGF2, ABCA1, COX2*, and *SMAD7*, have been identified as key progressor of T2DM-related atherosclerosis (84). Increased acetylation of histone 3 lysine 9 and histone 3 lysine 27 is seen in atherosclerotic plaques (85). Histone 3 lysine 9 acetylation in smooth muscles and macrophages is associated with greater severity of atherosclerosis (85). On the other hand, reduced methylation of the same histones is noted in such plaques.

Several miRNAs can be used as biomarkers of coronary artery disease (CAD). miR -126, -208a, -210, and -370 have shown promising diagnostic accuracy in CAD diagnosis among T2DM patients (86–88). Similarly, miR -195-5p and -451a have been used to detect transient and acute ischemic strokes in diabetic patients (89). miR -32-3p, -106-5p, -1246, and -532-5p have also been utilized as markers of acute ischemic strokes in the general population (90). These studies highlight the promising role of epigenetics as diagnostic markers for atherosclerosis in T2DM patients.

### 7.2 Diabetic retinopathy

Retinopathy is a significant diabetic consequence that can impair vision and lead to blindness. Diabetic retinopathy is associated with global DNA methylation, which is predictive of for the disease (odds ratio: 1.53) (91). Methylation of the promoter for MutL homolog 1, an enzyme responsible for repairing base mismatches in mtDNA, is increased in diabetic retinopathy, preventing the enzyme from correcting mtDNA damage (92). These effects, however, are reversed by administration of DNA methyltransferase-1 inhibitors. Increased histone 3 lysine 9 acetylation promotes diabetic retinopathy by activating the proinflammatory NF-κB pathway (93). Additionally, methylation of histone 4 lysine 20 at *SOD2* (superoxide dismutase-2) downregulates the antioxidant, exacerbating diabetic retinopathy and reinforcing metabolic memory of retinal cells (93). Several miRNAs have also been identified as potential biomarkers in the sera and plasma of diabetic retinopathy patients, such as miR -21, -27b, -126, and -190-5p, further solidifying the role of epigenetics in diabetic retinopathy (94).

### 7.3 Diabetic neuropathy

Diabetic neuropathy is the most common and debilitating complication of T2DM, present in more than one-third of diabetics (95–97). Low levels of DNA methylation is associated with diabetic neuropathy in T2DM patients (98). Recent studies have highlighted that diabetes mellitus effects genes crucial for nerve regeneration and functionality. For example, Gastoł et al. demonstrated that type 1 diabetics with neuropathy have greater methylation of *NINJ2* (ninjurin-2, responsible for nerve regeneration) and lower methylation of *BRSK2* (BR serine/threonine kinase-2, responsible for nerve functions) than diabetics without neuropathy (99). Reduced mtDNA copies has also been linked to diabetic neuropathy and is associated with the homozygous variant genotype for the polymorphism rs3746444 of *MIR499A* (100). Genetic variations in miR -146a, -128a, and -27 also increase susceptibility to diabetic neuropathy among T2DM patients (101).

Several miRNAs have fallen under the spotlight in patients with diabetic neuropathy. miR -33 and -380 are expressed in nociceptive neurons and are key mediators of diabetic pain (102). Circulating miRNAs, such as miR -199a-3p and -499A, could potentially be utilized to detect diabetic retinopathy and have been implicated in the disease (103). However, further studies are needed to determine their diagnostic accuracy in T2DM patients.

### 7.4 Diabetic cardiomyopathy

Diabetic cardiomyopathy is defined as myocardial disease in diabetic patients which cannot be attributed to any common cardiovascular etiologies or risk factors (104). The condition can be seen in 12% of diabetics and its prevalence is expected to rise exponentially in the coming years (105). The role of epigenetics in diabetic cardiomyopathy has been demonstrated by several studies. Tao et al. found that DNA methyltransferase-1 triggers cardiac fibroblast autophagy in diabetic cardiac fibrosis models by inhibiting the androgen receptor axis (106). Inhibiting the enzyme restores increases androgen receptor expression in these models. Promoter DNA demethylation of *KEAP1* is seen in the myocardium of diabetic patients (107). Kelch-like ECH-associated protein-1 (KEAP1) binds to NF-E2-related factor 2 (Nrf2), resulting in its proteasomal degradation (107). Nrf2 activates several antioxidant enzymes; hence, its degradation is key to diabetic cardiomyopathy progression. Expression of the protein liver X receptor-ɑ (LXRɑ) gradually increases with diabetic cardiomyopathy progression as a result of promoter demethylation (108). Chronic activation of LXRɑ induces β-cell apoptosis and, accordingly, predisposes to T2DM (109). Interestingly, LXRɑ improves myocardial glucose tolerance and blunts cardiac hypertrophy in T2DM mice (110). It could be possible that LXRɑ mediates protective effects in acute settings and eventually becomes pathogenic in more chronic settings. Further studies are required to demonstrate the role of LXRɑ and associated epigenetic modifications in diabetic cardiomyopathy. Epigenetics also influence cardiac cell cycle arrest in diabetic cardiomyopathy. Complete methylation of the cyclin D_1_ and complete demethylation of p21^WAF1/CIP1^ gene have been noted in early in the development of the condition (111).

In conclusion, epigenetic modifications are crucial mediators of T2DM-related comorbidities. Furthermore, a wide spectrum of circulating miRNAs can be used to predict and diagnose these conditions in this patient population. A comprehensive summary of the various epigenetic modifications involved in the pathogenesis of T2DM is presented in **Table 1**.

**Table 1:** Summary of key epigenetic modifications in T2DM pathogenesis and complications.

Condition	Epigenetic Modification	Key Findings	References
Atherosclerosis	DNA Methylation	Aberrant methylation of genes like SOD2 and FGF2 contributing to atherosclerosis in T2DM.	(84)
Diabetic Retinopathy	Histone Acetylation	Increased H3K9 acetylation activating NF-κB pathway in retinopathy.	(93)
Diabetic Neuropathy	DNA Methylation	Low levels of DNA methylation associated with neuropathy in T2DM patients.	(98)
Diabetic Cardiomyopathy	DNA Methylation	Methylation changes in genes like KEAP1 and LXRɑ affecting cardiomyopathy progression.	(107, 108)

## 8 Conclusion

Epigenetic research offers the potential to enhance T2DM prevention and treatment by unearthing novel molecular mechanisms responsible for the progression of the disease and its associated complications. Dysregulation of epigenetic modifications induce T2DM by promoting several cornerstones of the disease, such as obesity, IR, β-cell dysfunction, mitochondrial dysfunction, and cellular senescence. Additionally, lifestyle choices, including diet and exercise, also promote and reduce the risk of T2DM through epigenetic modifications. Preclinical studies have identified a wide range of therapeutic targets, such as DNA methyltransferase-1, with promising results. Clinical studies are needed to determine whether these studies can be translated to the human population. Additionally, further studies are needed to solidify the role of epigenetics in cellular senescence and mitochondrial dysfunction among T2DM patients.

## Author contributions

CJ: Writing – original draft. TA: Writing – original draft, Writing – review & editing. ZA: Writing – original draft. BS: Visualization, Writing – original draft, Writing – review & editing. AA: Writing – original draft. KA: Writing – review & editing. AY: Conceptualization, Supervision, Writing – review & editing.
